# Supplementary material for: Magnetic and MRI Contrast Properties of HumAfFt-SPIONs: Investigating Superparamagnetic Behavior and Enhanced T2-Weighted Imaging Performance
Source: Int J Mol Sci. 2025 Apr 9;26(8):3505. doi: 10.3390/ijms26083505 (PMC12027249; doi:10.3390/ijms26083505)
Supplement: Supplementary file 1 [file ijms-26-03505-s001.zip › ijms-3562385-supplementary.pdf]

SUPPLEMENTARY MATERIALS

Magnetic and MRI Contrast Properties of HumAfFt-SPIONs: Investigating Superparamagnetic Behavior and Enhanced T_2-Weighted Imaging Performance

Luisa Affatigato[1], Mariano Licciardi[2,*], Maria Cristina D'Oca[1], Luca Cicero[3], Alessandra Bonamore[4], Alessio Incocciati[4], Alberto Macone[4], Christian Dirk Buch[5], Stergios Piligkos[5], Alberto Boffi[4], Valeria Militello[1]

[1]Department of Physics and Chemistry – Emilio Segrè, University of Palermo, 90128 Palermo, Italy
[2]Department of Biological, Chemical and Pharmaceutical Sciences and Technologies (STEBICEF), University of Palermo, 90123 Palermo, Italy
[3]Istituto Zooprofilattico Sperimentale della Sicilia - A. Mirri, 90100 Palermo, Italy
[4]Department of Biochemical Sciences - A. Rossi Fanelli, Sapienza University, 00185 Rome, Italy
[5]Department of Chemistry, University of Copenhagen, DK-2100 Copenhagen, Denmark
*Corresponding author: mariano.licciardi@unipa.it; +393358086576

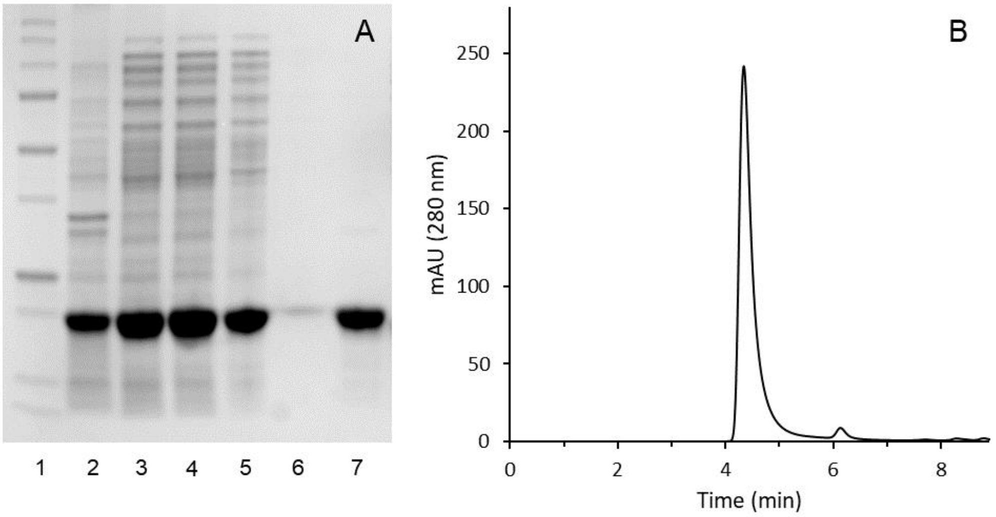

Figure S1. Characterization of HumAfFt. A) SDS-PAGE: 1) marker; 2) sonication pellet; 3) sonication supernatant; 4) 70% ammonium sulfate pellet; 5) heat treatment (78°C); 6) Post crossflow ultrafiltration; 7) post gel filtration). B) HP-SEC chromatogram of purified HumAfFt.

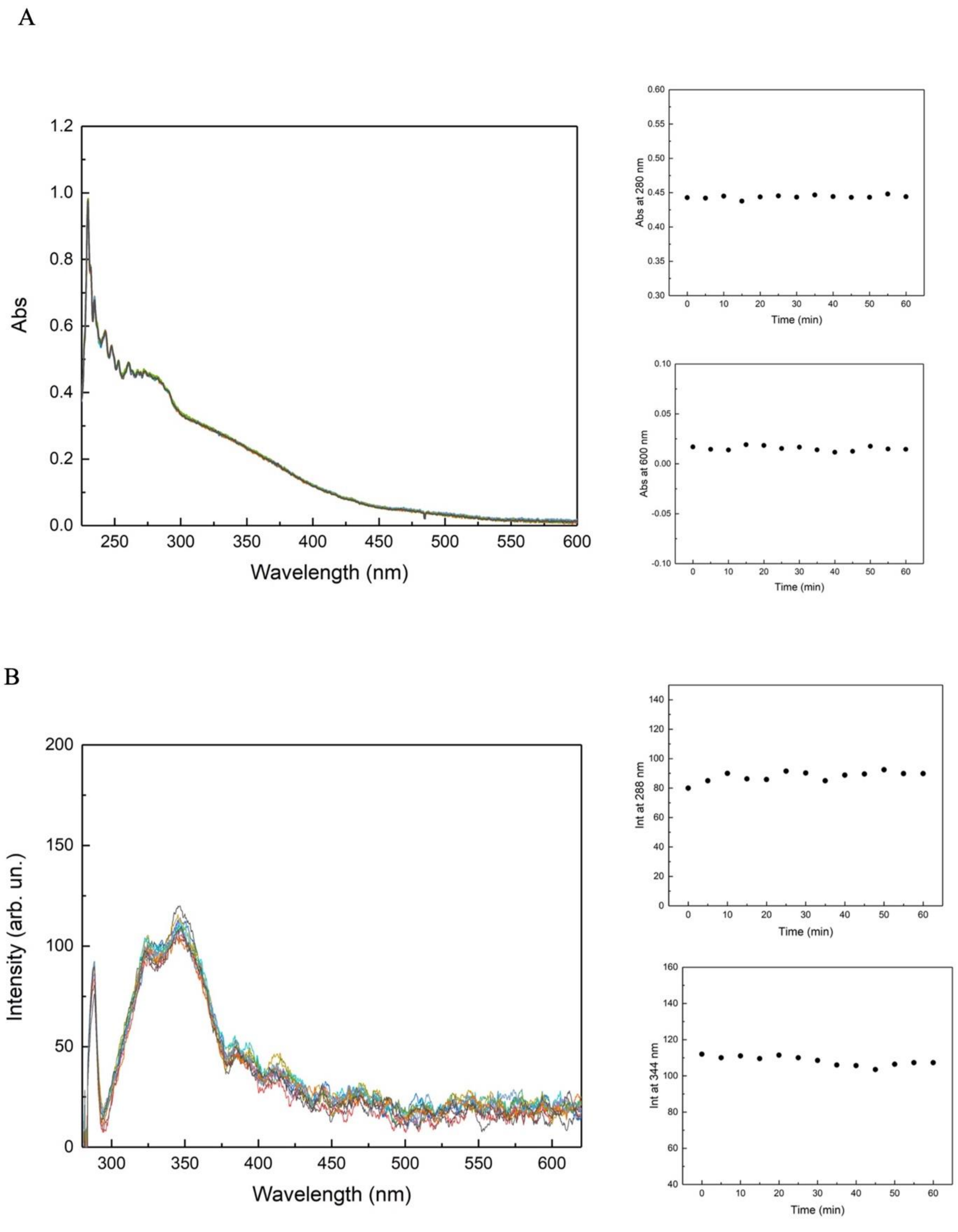

Figure S2. A) Absorption spectra of HumAfFt-SPIONs at different times (every five minutes for one hour) [HumAfFt]= 0.1 mg/mL; [SPIONs]= 0.01 mg/mL. The spectra on the right show the absorption at 280 nm and at 600 nm respectively. B) Fluorescence emission spectra of HumAfFt-SPIONs at different times (every five minutes for one hour) [HumAfFt]= 0.1 mg/mL; [SPIONs]= 0.01 mg/mL. The spectra on the right show the intensity at 288 nm and at 344 nm respectively.
